# Influenza vaccination coverage in pediatric population in Italy: an analysis of recent trends

**DOI:** 10.1186/s13052-022-01271-0

**Published:** 2022-05-16

**Authors:** Floriana D’Ambrosio, Teresa Eleonora Lanza, Rosaria Messina, Leonardo Villani, Angelo Maria Pezzullo, Walter Ricciardi, Aldo Rosano, Chiara Cadeddu

**Affiliations:** 1 Section of Hygiene, Department of Life Sciences and Public Health, Università Cattolica del Sacro Cuore, Rome, Italy; 2 National Institute for Public Policies Analysis (INAPP), Rome, Italy

**Keywords:** Influenza, Vaccination, Italy, Coverage, Children

## Abstract

**Background:**

Influenza is a major cause of morbidity, mortality and exacerbation of extant chronic disease worldwide. Influenza vaccination is thus fundamental to reduce the burden of disease. In this study, we describe the trend of influenza vaccination coverage in the seasons 2010/11–2020/21 among children aged < 2, 2–4 and 5–8 in Italy.

**Methods:**

We analyzed the trend of influenza vaccination coverage in the pediatric population in Italy from the 2010/11 to the 2020/21 season at national and regional level and observed the incidence of influenza-like illness (ILI) in the pediatric population between 2010/11 and 2020/21.

**Results:**

In the period 2010/11–2019/20 the highest value of coverage (4.5%) was reached in the age group 2–4 and 5–8 (season 2010/11 and 2011/12, respectively), while the lowest belonged to the < 2 group (1.1% in the season 2015/16). In the season 2020/2021 all the age groups reported a substantial increase of coverage compared with the previous season. The highest value (19.0%) was reported in the age group 2–4, followed by the group 5–8 and <  2 (13.1 and 9.2%, respectively). Considering the rates of annual ILI cases, the highest value for the 0–4 age group was 18.5% in the 2011/12 season; for the 5–14 age group, the highest value was 27.7% in the 2010/11 season.

**Conclusions:**

Over the past 11 years pediatric influenza vaccination coverage in Italy has been low, with relevant differences across regions and seasons, albeit a general increase in coverage has been observed in the 2020/21 season. Universal influenza vaccination for children should be considered as a priority for the high incidence in this age group. Further research is needed to improve knowledge and comparability of coverage rates, and to identify the best practices for organizational models of delivery which can support the improvement of trends, the acceptability and accessibility by parents and awareness in stakeholders and decision makers.

**Supplementary Information:**

The online version contains supplementary material available at 10.1186/s13052-022-01271-0.

## Introduction

Influenza is a major cause of morbidity, mortality and exacerbation of extant chronic disease worldwide, causing 1 billion cases, 3–5 million cases of severe illness and 290,000–650,000 deaths on average each year due to respiratory complications [1]. In Europe, seasonal influenza accounts for 4 to 50 million cases and up to 70,000 deaths every year [2]. As for Italy, an average of 8000 deaths from influenza and its complications are annually reported [3], while influenza-like illness (ILI) - defined as a syndrome that occurs in anyone (adults or children) presenting with sudden and rapid onset at least one general symptom (fever, malaise/exhaustion, headache, muscle aches) and at least one respiratory symptom, such as cough, sore throat, and shortness of breath - affects about 9% of the Italian population every year [4].

The clinical and economic burden of seasonal influenza varies in the general population and it is more relevant in vulnerable groups such as children, pregnant women, the elderly and people with chronic diseases [5]. It is estimated that this disease affects annually 20–30% of the pediatric population, due to the attendance of community settings like schools and educational and social centres and the immaturity of the immune system’s mechanisms, with the continuous antigenic drift of the virus that makes children particularly susceptible to this infection [6]. Mainly during the cold months, the pediatric population, and especially the children under 2 years of age who are furthermore unable to communicate their symptoms, has the highest risk of ILI and severe disease leading to hospitalization. As children shed the virus in greater amounts and for longer periods than adults of any age, they also represent one of the main causes of the spread of the infection in the community [7, 8].

Thus, influenza vaccination is fundamental to reduce the burden of disease. It has a direct protective effect on children, reducing hospitalizations [9] and risk of death [10], and it also affords indirect protection to close and susceptible contacts, like elderly [11]. Despite this, it is generally recommended only for children at risk of developing serious flu-related complications (i.e. affected by chronic diseases) and offered free of charge to all pediatric population only in some countries (i.e. USA, Canada, United Kingdom, Finland, Slovakia, Latvia) [12].

Influenza vaccination for children has become even more important during the SARS-CoV-2 pandemic [13], since influenza presents clinical symptoms similar to COVID-19 [14] and the coinfection is associated with worse clinical outcomes, with more than a doubled risk of mortality compared with people with COVID-19 [15]. Furthermore, since the COVID-19 vaccines are currently approved in Europe only for people aged ≥5 years [16], increasing the influenza vaccination coverage in younger children could mitigate the disease-specific burden and ease the differential diagnosis [17]. In order to achieve this aim, in 2020 the Italian Ministry of Health issued a Circular for the season 2020/21 recommending free influenza vaccination to children from 6 months to 6 years old [18]. Although the pediatric vaccination remains one of the most effective strategies to reduce the health and economic burden of influenza at population level [9, 19], the coverage rates among children remained very low over the years.

The aim of this study is to describe the trend of influenza vaccination coverage in the seasons 2010/11–2020/21 among children aged < 2, 2–4 and 5–8 in Italy and to evaluate the impact of COVID-19 pandemic on the vaccination coverage in the season 2020/2021, at national and regional level.

## Methods

### Data collection and statistical analysis

In this retrospective observational study, we analyzed the trend of influenza vaccination coverage in the pediatric population in Italy from the 2010/11 to the 2020/21 season at national and regional level. The incidence of ILI in the pediatric population was also taken into account. Data on vaccine coverage and ILI incidence were extracted from the official online reports of the Italian Ministry of Health [20] and of the Italian National Institute of Health with its integrated surveillance system called InfluNet [21], respectively. This system was established in 1999, coordinated by the Italian National Institute of Health, and it monitors influenza through a national network of about 1000 General Practitioners and family pediatricians (in charge of about 2% the Italian population) who report cases from individuals with ILI. It also collects samples from a subset of patients for virological confirmation. The estimation of the ILI incidence at population level is based on the samples collected through this integrated surveillance system.

Regarding the vaccination coverage, the analysis for each influenza season was focused on the pediatric population of the following age groups: < 2, 2–4, and 5–8 years old. We considered the rate change in coverage at regional level during the study period and the variation in terms of increase or decrease occurring during the season 2019/20 vs 2020/21 for each age group. Finally, we calculated the annual incidence of ILI in the age groups considered (0–4 and 5–14 years old) and the proportion of ILI cases in the age groups 0–4 and 5–14 years calculated on the basis of the samples collected through InfluNet surveillance system.

### Regional policy

The Italian Ministry of Health Circular “Influenza prevention and control: recommendations for the 2020/21 season” [18] extended free influenza vaccination to the pediatric population aged between 6 months and 6 years. In Italy, while the central government sets the fundamental principles and goals of the health system and determines the core benefit package of health services available to all citizens, the regions, that are the first-level constituent entities, are responsible for organizing and delivering primary, secondary and tertiary healthcare services, as well as preventive and health promotion services [22]. In order to evaluate which regions implemented the extension of free vaccination, we searched grey literature, institutional websites of Italian regions and autonomous provinces, of local health authorities and of relevant national medical societies and associations.

## Results

### Vaccination coverage in the pediatric population. Seasons 2010/11–2020/21

**Fig. 1:**
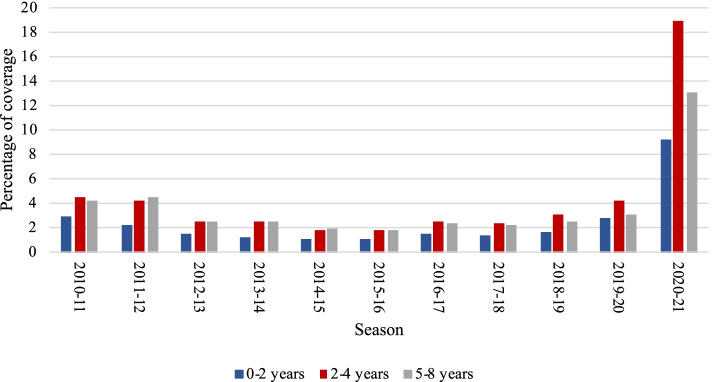
Influenza vaccination coverage rates in children by age group from 2010/11 to 2020/21

Influenza vaccination coverage in the Italian pediatric population showed variations during the last eleven seasons (Fig. 1). In the whole period considered, the coverage at national level in the < 2 age group has always been lower compared to the other groups (2–4 and 5–8 years), albeit it represents the pediatric population group with a significantly higher incidence of ILI every year.

In the period 2010/11–2019/20 the highest value of coverage (4.5%) was reached in the age group 2–4 and 5–8 (season 2010/11 and 2011/12, respectively), while the lowest belonged to the < 2 group (1.1% in the season 2015/16). All the three age groups reported the lowest values of coverage in the season 2015/16 (1.1, 1.8 and 1.8% in the age group < 2, 2–4, 5–8, respectively). Then, a gradual increase was observed until the 2019/20 season, whose values almost doubled those of 2015 (2.8, 4.2 and 3.1% in the age group < 2, 2–4, 5–8, respectively). However, concerning the period 2010/11–2019/20, a decrease in vaccination coverage was observed in all the age groups (− 0.1, − 0.3 and − 1.2% in the group < 2, 2–4 and 5–8, respectively).

In the season 2020/2021 all the age groups reported a substantial increase of coverage compared with the previous season. In particular, the highest value (+ 14.8%, coverage of 19.0%) was reported in the age group 2–4, followed by the group 5–8 and <  2 (+ 10%, coverage of 13.1% and + 6.4%, coverage of 9.2%, respectively). Considering the entire period, in each season the age group 2–4 registered the highest vaccination coverage compared with the other groups, with the exception of the years 2011/12, 2013/14, and 2014/15.

### Annual influenza-like illness (ILI) cases in Italy

The highest proportion of ILI confirmed cases among the 0–4 and 5–14 age group was reached in the seasons 2011/12 (18.48% on the total of ILI) and 2010/11 (27.72% on the total of ILI), respectively (Table 1). On the other hand, from 2010/11 to 2020/21, the seasonal estimated cumulative ILI incidence in the age group 0–4 years ranged from a minimum of 7.4% in the season 2020/21 to a maximum of 37.4% in the season 2017/18, and in the age class 5–14 years ranged from a minimum of 3.5% in the season 2020/21 to a maximum of 20.7% in the season 2017/18 (Table 2). Moreover, weekly peaks in the incidence of ILI cases were reached up to 41.0% in the 2017/18 season and 41.6% in the 2018/19 season for the 0–4 age group; considering the 5–14 age group, the peak was recorded in the 2019/20 season (27.7%). Interestingly, during the season 2020/21 the samples sent by the reporting doctors to the laboratories were 6818, but none of these tested positives for influenza strains (Fig. S1- See Additional file 1).

**Table 1:** ILI cases in pediatric age from 2010/2011 to 2020/2021

Seasons	Cases 0–4 years	Cases 5–14 years	Total cases	% 0–4 years	% 5–14 years
2010/2011	25,072	37,942	136,862	18.32	27.72
2011/2012	20,590	23,030	111,407	18.48	20.67
2012/2013	23,181	32,825	136,051	17.04	24.13
2013/2014	17,591	20,041	99,205	17.73	20.2
2014/2015	20,566	28,130	134,944	15.24	20.85
2015/2016	18,235	28,105	107,307	16.99	26.19
2016/2017	17,413	22,351	120,453	14.46	18.56
2017/2018	29,505	37,409	193,693	15.23	19.31
2018/2019	28,814	35,972	185,236	15.56	19.42
2019/2020	29,505	37,409	175,753	16.79	21.28
2020/2021	6043	6864	60,709	9.95	11.31

**Table 2:** Estimated seasonal incidence of ILI cases in pediatric and total population from 2010/2011 to 2020/2021 (data source: InfluNet – National Italian Institute of Health)

Seasons	0–4 years		5–14 years		Total population	
	Cases	Cumulative incidence per 1000	Cases	Cumulative incidence per 1000	N. cases	Cumulative incidence per 1000
2010/2011	706,000	25.1	1,106,863	19.7	5,871,936	9.8
2011/2012	618,979	22.1	717,792	12.7	4,948,074	8.2
2012/2013	703,156	25.5	1,049,457	18.7	6,072,854	10.1
2013/2014	561,252	20.6	671,322	11.8	4,635,259	7.7
2014/2015	656,545	24.5	966,924	16.9	6,300,683	10.4
2015/2016	585,098	22.5	930,605	16.3	4,989,719	8.2
2016/2017	555,761	22.0	719,671	12.6	5,438,485	9.0
2017/2018	915,245	37.4	1,163,306	20.7	8,604,314	14.3
2018/2019	859,487	36.3	1,071,916	19.3	8,020,797	13.4
2019/2020	725,557	31.6	1,088,897	19.8	7,509,361	12.6
2020/2021	165,297	7.4	190,696	3.5	2,400,842	4.0

### Regional transposition of the Italian Ministry of Health circular for the season 2020/21

In the context of the COVID-19 pandemic, the Italian Ministry of Health Circular “Influenza prevention and control: recommendations for the 2020/21 season” extended free seasonal influenza vaccination to healthy children from 6 months to 6 years old. Out of 21 Regions, 16 implemented the Circular. In particular, Piedmont, Emilia-Romagna, Tuscany, Calabria and Basilicata did not implement the circular and, while strongly recommending vaccination in the pediatric group, they kept offering it for free only to children belonging to categories at higher risk. In Emilia-Romagna, pediatric vaccination remained offered for a fee like the previous seasons. Marche and Apulia regions transposed the circular only partially, with Apulia extending the vaccination to healthy children aged between 6 months and 6 years for free depending on the availability of doses (Table S1- See Additional file 2).

### Comparison of regional vaccination coverage in the pediatric population between seasons 2019/20–2020/21

**Fig. 2:**
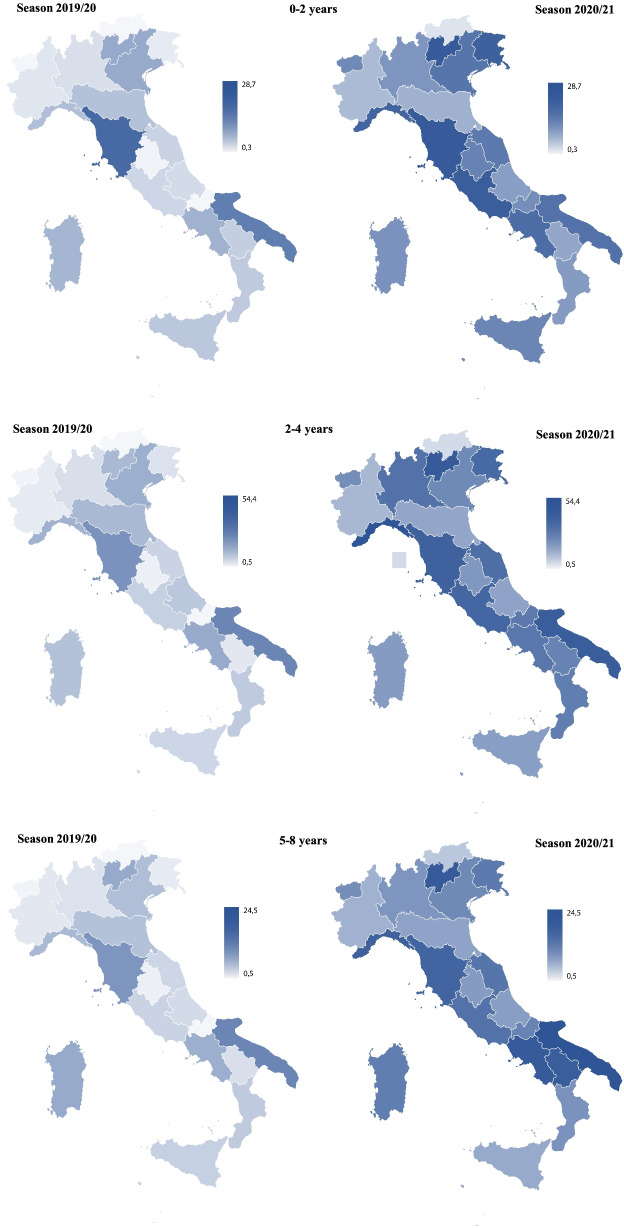
Influenza vaccination coverage rates in children by age group in the Italian regions: seasons 2019/2020 and 2020/2021

Vaccination coverage in the season 2019/20 and 2020/21 showed relevant differences among regions, in all the age groups. However, all the regions reported an increase in the season 2020/21 (Fig. 2).

Considering the < 2 age group, in the season 2019/20 Tuscany registered the highest value of coverage (14.3%), which almost double the second highest (Apulia, 7.5%). All the other regions had a coverage < 5%, with Umbria, Valle d’Aosta and Bolzano reporting the lowest ones (0.4, 0.3 0.3%, respectively). In the season 2020/21 Trento reported the highest value (28.7%), followed by Tuscany (16.4%), and Lazio (16.2%). Emilia-Romagna, Piedmont and Bolzano reported the lowest values, below 3% (2.6, 2.0 and 1.1%, respectively). Five regions reported an increase > 10% compared with the season 2019/20, with Trento registering the highest rise equal to 24.9%, while the increase was < 1% only in Emilia-Romagna and Bolzano (Table 3). Regions that did not implement the Circular (Piedmont, Emilia-Romagna, Tuscany, Basilicata and Calabria) reported a modest increase, below 3% (Table 3).

**Table 3:** Influenza vaccination coverage rates in the pediatric age (0–8 years) in 2019/2020 and 2020/2021 and relative variation

Region	< 2 years			2–4 years			5–8 years		
	2020/21	2019/20	variation	2020/21	2019/20	variation	2020/21	2019/20	variation
Piedmont	2.0	0.8	1.2	3.8	1.5	2.3	3.4	1.2	2.2
Valle d’Aosta	6.5	0.3	6.2	14.2	0.8	13.4	9.6	0.7	8.9
Liguria	15.1	1.8	13.3	54.4	4.2	50.2	19.4	2.9	16.5
Lombardy	5.3	1.1	4.2	22.1	1.9	20.2	15.5	1.4	14.1
Trento	28.7	3.8	24.9	31.9	3.5	28.4	17.4	3.3	14.1
Bolzano	1.1	0.3	0.8	2.0	0.5	1.5	2.2	0.5	1.7
Veneto	10.2	3.3	6.9	16.1	4.3	11.8	10.1	2.8	7.3
Friuli Venezia Giulia	15.9	0.7	15.2	25.3	1.7	23.6	12.1	1.1	11.0
Emilia-Romagna	2.6	1.8	0.8	6.5	3.5	3.0	6.0	2.7	3.3
Tuscany	16.4	14.3	2.1	27.9	13.5	14.4	17.8	9.2	8.6
Umbria	7.3	0.4	6.9	11.5	1.0	10.5	6.7	0.8	5.9
Marche	9.2	1.3	7.9	23.0	2.2	20.8	15.2	1.6	13.6
Lazio	16.2	1.3	14.9	26.5	2.3	24.2	15.4	1.7	13.7
Abruzzo	4.4	1.3	3.1	8.8	2.4	6.4	6.0	1.5	4.5
Molise	6.5	0.5	6.0	18.9	0.7	18.2	11.7	0.6	11.1
Campania	13.9	2.6	11.3	21.4	4.7	16.7	20.0	4.0	16.0
Apulia	12.1	7.5	4.6	30.3	16.2	14.1	24.5	10.5	14.0
Basilicata	3.6	1.4	2.2	17.9	1.5	16.4	19.8	1.4	18.4
Calabria	4.5	1.6	2.9	18.2	2.3	15.9	9.4	2.0	7.4
Sicily	7.2	1.7	5.5	8.8	2.1	6.7	5.0	1.8	3.2
Sardinia	6.2	2.2	4.0	8.8	2.8	6.0	11.7	4.9	6.8
**Italy**	**9.2**	**2.8**	**6.4**	**19.0**	**4.2**	**14.8**	**13.1**	**3.1**	**10.0**

In the 2019/20 season, the age group 2–4 years reported the highest vaccination coverage (4.2%), compared with the other groups. Apulia and Tuscany were the only regions with a coverage > 10% (16.2 and 13.5%, respectively), while Valle d’Aosta, Molise and Bolzano showed a coverage < 1% (0.8, 0.7 and 0.5%, respectively). In the season 2020/21, six regions (Liguria, Lombardy, Trento, Friuli Venezia Giulia, Marche and Lazio) improved the coverage of more than 20%, with the highest level of Liguria reaching 50.2%, while nine regions (Valle d’Aosta, Veneto, Toscana, Umbria, Molise, Campania, Apulia, Basilicata and Calabria) increased it from 10 to 20%. Only six regions reported a rise of less than 10%. Emilia-Romagna, Piedmont (which did not implement the Circular) and Bolzano showed the lowest increase (< 3%). In this context, 15 regions reached a coverage higher than 10% and nine regions reported a coverage higher than the national average (19.0%).

Finally, in the season 2019/20, Apulia, Tuscany and Sardinia reported the highest coverage (10.5, 9.2 and 4.9%, respectively) in the age group 5–8. On the contrary, Umbria, Valle d’Aosta, Molise and Bolzano had a value of less than 1%. As for the other age groups (< 2 and 2–4 years), an increase of coverage was observed in all regions in the season 2020/21. Indeed, ten regions reported a growth of more than 10% compared to the season 2019/20 (Basilicata, Liguria and Campania reported the highest increase, equal to + 18.4%, + 16.5 and + 16.0%, respectively), while Bolzano was the only region with an increase of less than 2%. Tuscany, Calabria, Emilia-Romagna and Piedmont (the regions that did not transport the Circular) increased the coverage of 8.6, 7.4, 3.3 and 2.2%, respectively (Table 3).

## Discussion

The present study describes the trend of pediatric influenza vaccination coverage rates in Italy at national and regional level across eleven seasons (2010/11–2020/21) and assess the impact of the COVID-19 pandemic on the variation of vaccination coverage between the seasons 2019/20–2020/21. Results showed that the coverage trend in Italy in the timeframe considered remains low, with relevant differences across regions and seasons, and a general increase in coverage in the last 2020/21 season, probably due to the higher willingness from parents to vaccinate their children to protect them from influenza and facilitate the differential diagnosis with COVID-19. Consistent differences were also observed across regions in the implementation of the Italian Ministry of Health Circular for the extension of the free influenza vaccination to healthy children aged between 6 months and 6 years. Finally, a reduction of ILI and the absence of identified influenza cases during 2020/21 season were also reported, likely determined by the massive use of face masks, hand hygiene and social distancing due to the implementation of containment measures related to COVID-19 [23]. Influenza vaccination is a key preventive measure in reducing the clinical burden of disease, in terms of cases, hospitalizations, and deaths, and its associated costs both in children and adults [24, 25]. These costs are composed of direct ones related to medical visits, hospitalizations and medicines, but also indirect ones such as loss of productivity, with psychological and social impact for parents who see their children ill [26]. Moreover, and especially in Italy, the burden of influenza is related to children who stay a long time with their grandparents and consequently these elderlies become at a high risk of getting influenza by their nephews. In this context and for the indirect protection of their caregivers, children are undoubtedly an important target for influenza vaccination [27]. Analyzing the whole period taken as a reference in our study (2010–2021), a non-linear trend over time in pediatric coverage can be observed and, despite some increases recorded in few seasons, it remains generally low. In detail, a progressive decrease was observed from 2010 to 2016, with a significant collapse in coverage in the 2014/15 and 2015/16 seasons. The reasons behind this collapse could not be identified with certainty, but inappropriate communication by media and healthcare workers around influenza vaccination could be associated to it. In particular, a national misinformation episode on influenza elderly vaccination happened in November 2014 could have had an impact also on vaccination for children. It refers to a suspension as a precautionary measure by the Italian Medicines Agency (AIFA) of the use of two batches of an influenza adjuvate vaccine after three post-vaccination deaths reported through the Network of Pharmacovigilance and initially associated to the vaccination [28]. Unfortunately, the media did not emphasize the issue of European Medicines Agency (EMA) Pharmacovigilance Risk Assessment Committee statement denying the association and the impact of this misinformation in the general population remained so consistent that had a relevant aftermath also on the following influenza vaccination season [29].

After the two critical seasons of 2014/15 and 2015/16, a gradual increase in coverage was observed, reaching in the last 2020/21 season significantly higher values, in contrast with the results by Fogel et al. [30], who found a decline in influenza vaccination rates in children during the COVID-19 pandemic, probably due to lack of confidence and inconvenience by parental intent to obtain the influenza vaccine or introduced barriers to healthcare access. As initially mentioned, reasons for the increase seen in Italy could be instead attributed to a greater attention and sensitivity to respiratory infectious diseases preventable with vaccination derived during the COVID-19 pandemic. In a timeframe when COVID-19 vaccination was not available to the general population yet, the importance of allowing differential diagnosis with respiratory infections other than the one caused by SARS-CoV-2 was essential at every age. Indeed, for this reason the Italian Ministry of Health extended the active and free offer of influenza vaccination to the pediatric population aged 6 months to 6 years and no longer only to the risk categories. However, unfortunately this kind of recommendation has not been renewed in the Italian Ministry of Health Circular for the current season 2021/22, and only some Regions (e.g. Lazio) have kept the gratuity anyway [31, 32], contributing to the increase of inequalities in healthcare and in the vaccination offer in the country [33, 34]. Another important aspect of the present study deals actually with the regional differences regarding pediatric coverage, highlighted in the 2019/20 season but that seem noticeable also in the last examined season (2020/21). Despite an increase in recent years, vaccination coverage among all the Italian regions is still rather low. In general, the most performant Regions were Tuscany and Apulia, while Liguria was able to increase the vaccination coverage to 50% only in the age group 2–4. Evidence for these differences is not clearly found in literature, but it is supposed that the network of family pediatricians and their organizational aspect in these Regions are more efficient than in others [35]. For example, the opening of their clinics in the weekends or for additional hours during the week in order to vaccinate as many children as possible could be reasons for this phenomenon. Other examples to be considered aiming at increasing vaccination coverage are special events where vaccination is combined with health education and promotion activities facilitated by a multidisciplinary team of doctors, nurses, and cultural mediators, as carried out in Emilia Romagna region in some integrated primary care centers [36]. This kind of activities allows greater proximity to the local community as well. On the other hand, among the Regions with the lowest coverage rates there is the autonomous province of Bolzano, where the population confirmed to be unwilling to vaccinate especially for the pediatric age [37], despite the local government commitment to promote vaccination (i.e. extending influenza vaccination for free to the entire population). Looking at the European scenario, the comparison of Italian national influenza vaccination trends with other countries is a difficult matter. Data are in fact often unavailable for most countries and, if available, not updated. Exception is made by the United Kingdom, where since 2012 began the phased roll-out of the national influenza vaccination programme to ultimately cover all 2–16 years old children in the country and coverage rates currently reached over 60% of the target population [38, 39]. Data updated to 2018 are available also for other few countries of the WHO European Region: Estonia, Latvia, Poland Slovakia, and Slovenia, which reported low rates of vaccination coverage similarly to Italy (4.9, 3.1, 0.85, 1.4 and 0.1%, respectively), and Belarus, Finland, Israel, Malta and Russia, which presented a vaccination coverage > 40% (75.4, 42.5, 18.4, 55.0 and 57.4%, respectively) [38]. A possible explanation of the different coverage levels might be partly due to the heterogeneity of recommendations from a country to another, making in any case hard the comparative assessment [40–42]. As of 2015, indeed, only eight European countries (i.e. Austria, Finland, Latvia, Malta, Poland, Slovakia, Slovenia and the United Kingdom) recommend influenza vaccination for children, and only Finland, Latvia and the United Kingdom provide it free of charge [42].

Our study is the first in Italy to analyze regional vaccination coverage and strategies in the pediatric population. Vaccination coverage represents the best available indicator of vaccination strategies, as they provide information on their actual implementation in the area and on the efficiency of the vaccination system. Furthermore, the investigation on the differences of the Italian regions highlights implications related to organizational aspects, even if evidence about these is limited and should be improved. The present study has also the strength of using complete national real-world data, even if their aggregated form impedes quantitative analysis on the factors associated with decreasing or increasing trends. Finally, the scarce availability of data, particularly those related to the regional organizational domain, limited a complete analysis of the topic addressed in the study.

## Conclusions

The present study shows that in Italy influenza vaccination coverage in the pediatric population remain low, despite its clinical importance recently emphasized in the scientific literature, with benefits outweighing risks, and added value deriving from the improvement of the quality of life in children themselves and in their families, besides less expenditures in terms of access to Emergency Department, hospitalizations and outpatient visits [43, 44]. As underlined by many Italian medical scientific associations in the Schedule for Lifetime (“Calendario per la vita”) since 2014 [45, 46], universal influenza vaccination for children, at least in the age group 6 months-6 years, should be considered as a priority for the high incidence in this age group and their main role for the transmission of the virus in the population. Further research in this area is needed in order to improve knowledge and comparability of coverage rates, to identify the best practices for organizational models of delivery which can support the improvement of trends, the acceptability and accessibility by parents and awareness in stakeholders and decision makers.

## Supplementary Information

**Additional file 1: Figure S1.** Annual influenza-like illness (ILI) cases per 100,000 in children in Italy by age group.

**Additional file 2: Table S1.** Regional sources relating to the implementation of the Circular of the Italian Ministry of Health 2020/21 “Influenza prevention and control: recommendations for the 2021-2022 season”.

## Data Availability

All data generated or analysed during this study are included in this published article and its supplementary information files.

## Abbreviations

AIFA: Agenzia Italiana del Farmaco (Italian Medicines Agency)
COVID-19: Coronavirus Disease 2019
EMA: European Medicines Agency
ILI: Influenza-like Illness
SARS-CoV-2: Severe Acute Respiratory Syndrome Coronavirus 2
