# Supplementary material for: Influenza vaccination coverage in pediatric population in Italy: an analysis of recent trends
Source: Ital J Pediatr. 2022 May 16;48:77. doi: 10.1186/s13052-022-01271-0 (PMC9109451; doi:10.1186/s13052-022-01271-0)
Supplement: Supplementary file 2 — Additional file 2: Table S1. Regional sources relating to the implementation of the Circular of the Italian Ministry of Health 2020/21 “Influenza prevention and control: recommendations for the 2021-2022 season”. [file 13052_2022_1271_MOESM2_ESM.pdf]

Table S1. Regional sources relating to the implementation of the Circular of the Italian Ministry of Health 2020/21 "Influenza prevention and control: recommendations for the 2021-2022 season".

Region	Source
Piedmont	https://www.regione.piemonte.it/web/temi/sanita/stagione-influenzale-2020-2021
Valle d'Aosta	http://www.ausl.vda.it/notizie.asp?id=778&l=1&n=1247
Liguria	http://www.asl3.liguria.it/campagna-antinfluenzale-2020-2021.html
Lombardy	https://www.wikivaccini.regione.lombardia.it/wps/portal/site/wikivaccini/DettaglioRedazionale/le-vaccinazioni-quali/campagna-2020-2021
Trento	https://www.ufficiostampa.provincia.tn.it/Comunicati/Influenza-iomivaccino-al-via-la-campagna-di-vaccinazione
Bolzano	https://www.provincia.bz.it/news/it/news.asp?news_action=4&news_article_id=644310
Veneto	https://www.aulss3.veneto.it/Campagna-antinfluenzale
Friuli Venezia Giulia	https://www.regione.fvg.it/rafvg/cms/RAFVG/salute-sociale/promozione-salute-prevenzione/FOGLIA33/
Emilia-Romagna	https://www.regione.emilia-romagna.it/notizie/2020/ottobre/ancor-piu-prevenzione-covid-in-emilia-romagna-test-sierologici-gratuiti-in-farmacia-per-studenti-genitori-e-familiari-universitari/campagnadivaccinazioneantinflu.pdf
Tuscany	https://www.vaccinarsintoscana.org/notizie/2020/10/vaccinarsi-contro-linfluenza-al-via-la-campagna-vaccinale-della-regione-toscana
Umbria	https://www.regione.umbria.it/documents/18/24838485/Linee+di+indirizzo++vaccinazione+antinfluenzale+e++antipneumococco_+2020-2021.pdf/a0ef29c2-8dba-4b46-83e8-a3a1cf1913ea
Marche	https://www.vaccinarsinellemarche.org/vaccinazioni-marche/campagne-vaccinali-delle-marche/campagna-antinfluenzale-2020-2021
Lazio	https://www.salutelazio.it/vaccinazione-antinfluenzale-2020/21
Abruzzo	https://www.vaccinoantinfluenzaleabruzzo.it
Molise	http://www3.regione.molise.it/flex/cm/pages/ServeBLOB.php/L/IT/IDPagina/17572
Campania	http://www.aiopcampania.it/public/normativa/7906b2ba132b4ef3a36cdfe6fddec277.pdf
Apulia	https://www.vaccinarsinapulia.org/notizie/2020/07/antinfluenzale-stagione-2020/21.-raccomandazioni-ufficiali,-documento-del-nitag-e-appello-degli-igienisti<br>http://www.quotidianosanita.it/scienza-e-farmaci/articolo.php?articolo_id=87169<br>https://press.regione.apulia.it/-/avvio-campagna-di-vaccinazione-antinfluenzale-2020-2021
Basilicata	https://www.aspbasilicata.it/wp-content/uploads/DDG513_2020.pdf<br>https://www.lagazzettadelmezzogiorno.it/news/potenza/1253004/basilicata-una-colletta-per-avere-il-vaccino-contro-l-influenza.html
Calabria	https://portale.regione.calabria.it/website/portalmedia/decreti/2020-05/Ordinanza%20P.G.R.%20n.%2047_2020.pdf
Sicily	https://www.vaccinarsinsicilia.org/notizie/2020/08/tante-novit%C3%A0-della-campagna-vaccinale-antinfluenzale-2020/21#:~:text=Per%20tutte%20le%20ragioni%20sopracitate,rispetto%20alla%20stagione%202019%2F2020
Sardinia	https://www.aslcagliari.it/index.php?xsl=7&s=70944&v=2&c=288<br>https://www.vaccinarsinsardegna.org/it/notizie/2020/07/vaccinazione-anti-influenzale-2020-novit%C3%A0
