# Supplementary material for: Influenza vaccination coverage in pediatric population in Italy: an analysis of recent trends
Source: Ital J Pediatr. 2022 May 16;48:77. doi: 10.1186/s13052-022-01271-0 (PMC9109451; doi:10.1186/s13052-022-01271-0)

Figure S1. Annual influenza-like illness (ILI) cases per 100,000 in children in Italy by age group.

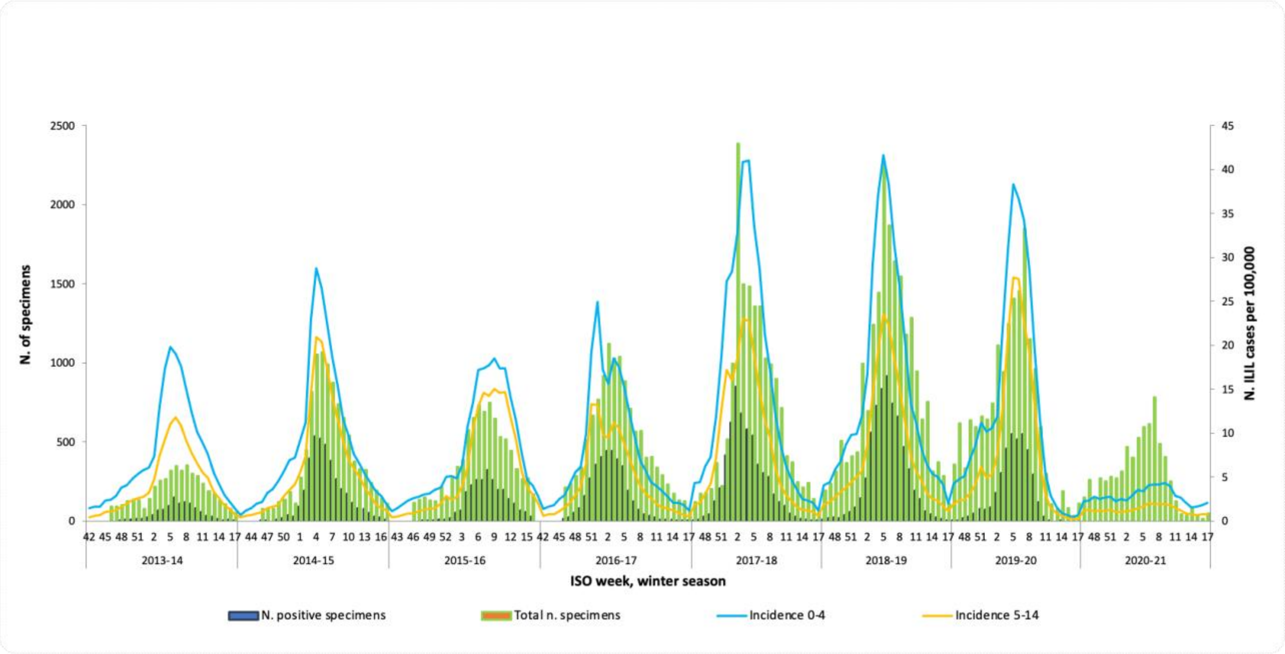

Supplement: Supplementary file 1 — Additional file 1: Figure S1. Annual influenza-like illness (ILI) cases per 100,000 in children in Italy by age group. [file 13052_2022_1271_MOESM1_ESM.pdf]
